# Antioxidants Differentially Regulate Non‐Photochemical Quenching

**DOI:** 10.1111/pce.70672

**Published:** 2026-06-15

**Authors:** Aline de Rodrigues de Queiroz, Jeremy H. Brown, Nicole R. Buan, Julie M. Stone, Katarzyna Glowacka, Rebecca L. Roston

**Affiliations:** ^1^ Department of Biochemistry University of Nebraska‐Lincoln Lincoln Nebraska USA; ^2^ Nebraska Centre for Plant Science Innovation University of Nebraska‐Lincoln Lincoln Nebraska USA; ^3^ Nebraska Centre for Redox Biology University of Nebraska‐Lincoln Lincoln Nebraska USA; ^4^ Institute of Plant Genetics, Polish Academy of Sciences Poznań Poland

**Keywords:** arabidopsis, ascorbic acid, glutathione, melatonin, oxidative stress, photosynthesis, xanthophylls

## Abstract

Non‐photochemical quenching (NPQ) dissipates excess light energy as heat, limiting reactive oxygen species production during photosynthesis. Exogenous antioxidants are widely reported to improve plant performance under stress, yet their impacts on NPQ are inconsistent across studies, complicating mechanistic interpretation. Here, we systematically compared six antioxidants – ascorbate, chitosan, coenzyme M, reduced glutathione, melatonin, and N‐acetyl cysteine – applied to *Arabidopsis thaliana* seedlings using individual concentration gradients, under standardised pH and illumination regimes. Antioxidants produced strong, divergent, and context‐dependent shifts in NPQ kinetics. Coenzyme M most strongly suppressed NPQ, decreasing induction rate, maximum, and relaxation rate, whereas melatonin and N‐acetyl cysteine generally enhanced NPQ maximum or relaxation and ascorbate uniquely accelerated NPQ induction. These effects changed at longer times post application. Using *psbs*, *vde* and *zep* null mutants, we show that short term ascorbate and coenzyme M NPQ effects require canonical NPQ components. The major contributor to their effects were opposing regulation of the xanthophyll cycle: ascorbate enhanced, while coenzyme M suppressed, light‐driven violaxanthin de‐epoxidation and altered xanthophyll flux during recovery. These data demonstrate two antioxidant‐specific mechanisms, confirming their specificity.

## Summary statement

In *Arabidopsis thaliana*, six antioxidants strongly and differentially reprogramme non‐photochemical quenching kinetics in a compound‐specific and concentration‐, time‐, and light‐dependent manner. Ascorbate enhances xanthophyll‐cycle de‐epoxidation, while coenzyme M suppresses zeaxanthin formation.

## 1 Introduction

Antioxidants are low molecular weight compounds or enzymes that counteract oxidation by balancing redox status and preventing oxidative stress in the cell, and as such, they are an essential part of cellular communication (Noctor et al. 2018; Sies 2020). Antioxidants have a great diversity in their chemical structures, redox capacities, subcellular localisations, mechanisms of action, and biosynthesis and catalysis rates (Noguchi et al. 2000; Foyer and Shigeoka 2011; Cannea and Padiglia 2025). Despite their diversity, antioxidants can have similar effects on plants when applied exogenously. Some of those effects include improving plant growth and optimising stress responses (Rodrigues de Queiroz et al. 2023; Brown et al. 2025).

Plants are prone to oxidative stress in response to external abiotic and biotic stressors, as well as the intrinsic stress of photosynthesis. Photosynthesis produces high levels of overoxidation and overreduction due to the inherent presence of oxygen and the generation of strong reductants in the electron transport chain (Scheibe et al. 2005). Therefore, plants have evolved sophisticated mechanisms to prevent redox imbalance during photosynthesis, including non‐photochemical quenching (NPQ, González et al. 2021). NPQ is a major photoprotective mechanism that dissipates excess absorbed light energy as heat, decreasing the quantum yield of PSII and limiting the formation of toxic reactive oxygen species (Müller et al. 2001; Murchie and Ruban 2020). NPQ is therefore crucial for high‐light acclimation, adaptation to fluctuating light, and overall fitness and survival (Wang et al. 2017; Yang et al. 2022; Schiphorst et al. 2023; Zuo et al. 2025). However, when light intensity decreases, slow NPQ relaxation can reduce photosystem II quantum yield and cause transient energy losses. Consistent with this, accelerating NPQ relaxation has been shown to enhance growth and yield in multiple crops (Hubbart et al. 2012; Kromdijk et al. 2016; Cowling et al. 2022; De Souza et al. 2022).

NPQ is stimulated by the buildup of the proton gradient across the thylakoid membrane, and full activation requires protonation of PSII subunit S (PsbS), and conversion of the xanthophyll pigments violaxanthin to zeaxanthin via the intermediate antheraxanthin (Yamamoto et al. 1962; Li et al. 2000; Ruban 2016; Demmig‐Adams et al. 2020). PsbS modulates NPQ sensitivity to low pH, while zeaxanthin acts as an allosteric regulator needed for full NPQ activation. (Xu et al. 2015). PsbS is a structural protein located in the thylakoid membrane, it has two lumen‐exposed loops containing two pairs of symmetrically arranged glutamate residues that are likely to be responsible for detecting pH changes (Li et al. 2004). In the dark, dimeric PsbS interacts with the light harvesting complex II (LHCII) bound to photosystem II (PSII). Under excess light, lumen acidification promotes PsbS monomerization, leading to detachment of LHCII from the PSII core and exposing energy dissipation sites (Correa‐Galvis et al. 2016). The xanthophyll cycle enzymes, violaxanthin de‐epoxidase (VDE) and zeaxanthin epoxidase (ZEP), regulate zeaxanthin abundance. Under excess light and low lumen pH, VDE reversibly converts violaxanthin to antheraxanthin and zeaxanthin using ascorbic acid as a substrate (Jahns et al. 2009). When zeaxanthin accumulates through stress or genetic manipulation, NPQ induction rate and NPQ maximum increase while relaxation slows (Niyogi et al. 1998; Kalituho et al. 2007a; Kalituho et al. 2007b; Haupt and Glowacka 2024). Under dark conditions and neutral pH, ZEP converts zeaxanthin back to violaxanthin (Jahns et al. 2009; Goss and Latowski 2020). VDE is redox‐regulated at its cysteine‐rich domain (Arnoux et al. 2009; Gollan and Aro 2020), and ZEP activity is also modulated under oxidative conditions (Bethmann et al. 2019; Holzmann et al. 2022).

Exogenous application of antioxidants is known to impact NPQ, particularly under stress. However, previous findings show divergent NPQ effects (reviewed in Rodrigues de Queiroz et al. 2023), likely due to variations in plant species, application methods, and experimental conditions. Here we systematically tested the effects of six antioxidants applied with individual concentration gradients on the NPQ kinetics of *Arabidopsis thaliana* under standardised conditions. We sought to determine whether antioxidant effects are general, acting through a shared pathway, or specific to each compound. We initially hypothesised that antioxidants would uniformly reduce NPQ by creating a more reduced cellular environment that inhibits VDE activity. Contrary to this expectation, our results reveal that antioxidants exert highly specific impacts on NPQ, though certain compounds can be grouped by their effects. Notably, we demonstrate that the modes of action for ascorbate and coenzyme M are driven specifically by xanthophyll cycle modifications. These findings suggest that rather than a universal inhibitory effect, different antioxidants operate through distinct and specialised molecular mechanisms.

## 2 Materials and Methods

### 2.1 Plant Material and Growth Conditions

*Arabidopsis thaliana* (Col‐0) seeds were surface‐sterilised for 30 min in 30% (vol/vol) bleach, rinsed five times with autoclaved water, and sown in 15‐cm Petri dishes (Sarstedt, 82.1184.500) containing 80 mL of half‐strength MS media with vitamins, without glycine (Caisson Labs, MSP02), 1% (wt/vol) sucrose, 0.5% (wt/vol) MES (2‐morpholinoethanesulfonic acid), adjusted to pH 5.7 with KOH, and solidified with 0.6% (wt/vol) Agargel (Sigma, A3301). Seeds were stratified in the dark for 3 days at 4°C, then placed in a growth chamber (Percival CU36L4) at 22°C (light period) and 18°C (dark period) under a 16 h light/8 h dark photoperiod for 12 days. Lighting was provided by T8 fluorescent bulbs at the level of 120 µmol photons m^−2^ s^−1^. As indicated, the following T‐DNA insertion mutants causing loss of function (grown identically) were also used: *vde/npq1* (AT1G08550; stock CS3771), *zep/aba1* (AT5G67030; stock CS3772), and *psbs/npq4* (AT1G44575; stock CS66021). For the proton flux measurements, protein, and pigment quantifications, plants were grown for 3 weeks in a 10:1 (vol/vol) mixture of Sungro Propagation Mix and Turface at 22°C (light period) and 18°C (dark period) under a 16 h light/8 h dark photoperiod. Every experiment consisted of a minimum of three independent growth trials. The total number of biological replicates varied with experiment and is indicated in figure legends for clarity.

### 2.2 Antioxidant Applications

Antioxidant solutions were always prepared fresh from dry stocks in 10 mM phosphate buffer, adjusted to pH 5.1. Phosphate buffer was chosen for its low reactivity to monovalent ions including common counter‐ion sodium, and strong pH buffering capacity. The antioxidants tested were l‐ascorbic acid (Research Products International A50040‐250), poly(d‐glucosamine) (chitosan; MP Biomedicals 150597), sodium 2‐mercaptoethanesulfonate (coenzyme M; AstaTech 46862), reduced γ‐l‐glutamyl‐l‐cysteinyl‐glycine (reduced glutathione; Acros Organics 120000250), N‐acetyl‐5‐methoxytryptamine (melatonin; Tokyo Chemical Industry M1105), and N‐acetyl‐l‐cysteine (Cayman Chemicals 20261). All antioxidant solutions were fully soluble with no visible precipitate throughout the application window. Antioxidant or control buffer (10 mM phosphate, pH 5.1) was applied by foliar spray with physical barriers to prevent overspray. Plants were sprayed until saturated, resulting in application of 8–10 μL per 12–day–old seedling. Unless otherwise noted (e.g., time‐course experiments), plants were dark‐incubated for 30 min at 22°C after spraying and then exposed to high light to activate NPQ as described below. Initial high concentrations of each antioxidant were determined from available literature on their application, selecting higher levels from all reported to prioritise detectable changes: ascorbate (Bulley et al. 2021), chitosan (Kowalski et al. 2006), coenzyme M (Brown et al. 2025), glutathione (Zeng et al. 2021), melatonin (Bajwa et al. 2014), and N‐acetyl‐cysteine (Yuce et al. 2024).

### 2.3 Chlorophyll Fluorescence Measurements

**Figure 1:**
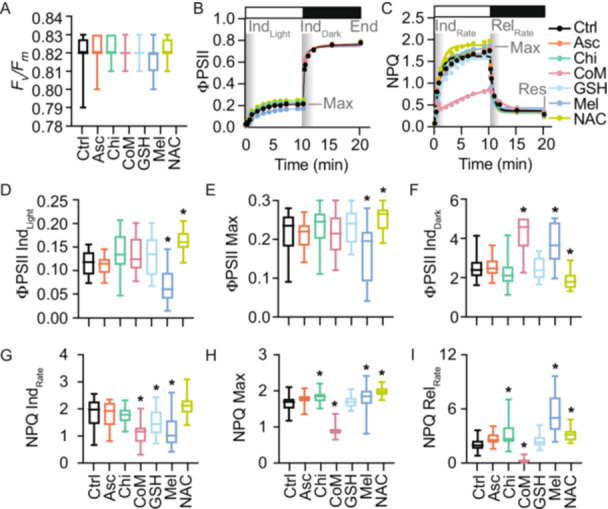
Differential effects of applied antioxidants on photosynthetic fluorescence parameters. 12‐day‐old *Arabidopsis thaliana* plants were treated with pH‐buffered phosphate (control), or antioxidants at final concentrations of 25 mM ascorbic acid (Asc), 1 g/L chitosan (Chi), 25 mM coenzyme M (CoM), 25 mM reduced glutathione (GSH), 0.25 mM melatonin (Mel), or 0.25 mM N‐acetyl cysteine (NAC). Seedlings were dark‐adapted for 30 min, then subjected to a pulse‐amplitude modulated (PAM) protocol − 10 min of illumination (white bar) followed by 10 min of darkness (black bar). A, Maximum quantum yield of PSII (*F*_*v*_ */F*_*m*_). B, Kinetics of operating efficiency of Photosystem II (ΦPSII). Filled circles indicate individual data points, lines indicate exponential fit curves, error bars indicate standard error of the mean. Grey gradient bars and grey text indicate kinetic parameters quantified in D – F. Rate of ΦPSII induction during illumination (Ind_Light_), ΦPSII at the end of the light protocol (Max), Rate of ΦPSII induction in darkness (Ind_Dark_), ΦPSII at the end of PAM protocol (End). C, Kinetics of non‐photochemical quenching (NPQ). Filled circles indicate individual data points, lines indicate hyperbolic fit curves, error bars indicate standard error of the mean. Grey gradient bars and grey text indicate kinetic parameters quantified in G – I. Induction rate of NPQ during illumination (Ind_Rate_), Maximum NPQ (Max), Relaxation rate of NPQ during darkness (Rel_Rate_), Residual NPQ at the end of PAM protocol (Res). Residuals had no significant differences among applications. D, ΦPSII Ind_Light_; E, ΦPSII Max; F, ΦPSII Ind_Dark;_ G, NPQ Ind_Rate;_ H, NPQ Max; I, NPQ Rel_Rate_. The data in all panels represent the same 24 biological replicates from three independent growth trials. Panels A and D – I are represented by box plots; box indicates 25th to 75th percentiles, and the whiskers show the minimum and maximum values. Statistically significant differences between control and applications by Dunnett's Post Hoc Tests are indicated with asterisks (*p*‐value < 0.02).

2 h after lights on, each seedling was gently transferred from the growth medium to one of the 32 central wells of a 96‐well plate to evenly space plants and ensure uniform illumination; damp sponges added in each well prevented desiccation and provided support. Antioxidants were applied by foliar spray, then dark‐incubated for 30 min at 22°C (unless otherwise noted). Chlorophyll fluorescence was recorded with a Closed FluorCam FC 800‐C imager (Photon Systems Instruments, Drasov, Czech Republic) using a pulse‐amplitude modulated protocol (PAM). Saturating pulses (2400 µmol photons m^−2^ s^−1^, 6500 K, 800 ms) were applied during both light and dark periods. After measuring *F_0_* in dark‐adapted plants, steady‐state fluorescence (*F_s_*) and maximum fluorescence were measured under actinic light (*F_m_′*) at 0, 0.26, 0.41, 0.745, 1.08, 2.08, 3.08, 4.08, 5.08, 6.08, 7.08, 8.08, 9.08, 10.08, 10.26, 10.41, 10.91, 11.91, 15.08, and 20.08 min. Actinic illumination was 800 µmol photons m^−2^ s^−1^ (a combination of 400 μmol m^−2^ sec^−1^ of a red‐orange light with λ_max_ = 617 nm and 400 μmol m^−2^ sec^−1^ of a cool white 6500 K light) for 10 min followed by 10 min darkness (recovery). Maximum quantum yield of PSII was defined as *F*_*v*_ */F*_*m*_ of dark‐adapted plants. NPQ was calculated as *F*_*m*_/(*F*_*m*_ *’*−1) (Bilger and Björkman 1994). ϕPSII and NPQ measurements were fitted to hyperbola to estimate the induction rate from the initial slope in the light, and the relaxation rate from the initial slope in the dark (Sahay et al. 2023). Maximums and end or residual points were defined from the last point in the light or dark, as indicated in Figure 1.

### 2.4 Proton Motive Force and Proton Flux Measurements

Total electrochromic shift was estimated on the youngest, fully expanded leaf of 3‐week‐old, soil‐grown Arabidopsis after 30 min of dark adaptation, followed by 5 min of 1000 µmol photons m^−2^ s^−1^ of light, using a MultispeQ V 2.0 (PHOTOSYNQ). For all measurements, the leaf fully covered the measurement aperture. The RIDES 2.0 protocol was used (Kuhlgert et al. 2016). Calculations of electrochromic shift, proton conductivity, and proton motive force were done on the PhotosynQ online platform as described previously (Sahay et al. 2024).

### 2.5 Protein Abundance and Photosynthetic Pigment Quantifications

Three‐week‐old soil‐grown plants were sprayed with antioxidant or control solutions and subsequently dark‐adapted for 30 min, exposed to 10 min at 1000 µmol photons m^−2^ s^−1^, and allowed 10 min dark recovery at 22°C. Samples were collected at three time points (post‐dark adaptation, 5 min high light, and 10 min dark recovery), flash‐frozen, homogenised, and split into 50 mg aliquots for protein and pigment analyses.

For protein analysis, 50 mg tissue was extracted in 500 µL clear Laemmli sample buffer (Bio‐Rad), clarified by centrifugation, heated 10 min at 98°C, and quantified by Bradford assay (Bio‐Rad). Equal amounts of total protein (10, 20 and 30 µg) were resolved by SDS‐PAGE (10% VDE and ZEP; 15% PsbS), transferred onto PVDF membranes, stained with Ponceau S, blocked in TBS with 1% Tween‐20% and 5% non‐fat dry milk (Bio‐Rad manual), and probed overnight with primary antibodies against the following proteins: PsbS (1:1000; Agrisera AS09533), VDE (1:1000; Agrisera AS15 3091), and ZEP (1:2500; Agrisera AS15 3092). Secondary antibody was goat anti‐rabbit IgG (H + L) (Invitrogen, 1:5000). Bands were visualised by chemiluminescence (Clarity Western ECL, Bio‐Rad) on a LI‐COR Odyssey Fc, and quantified using FIJI software (Schindelin et al. 2012).

For xanthophyll cycle pigment analysis, 50 mg tissue was extracted and analysed by HPLC at the Centre for Proteomics and Metabolomics at the University of Nebraska‐Lincoln (Vu & Alvarez, 2021). De‐epoxidation state (DEPS) was calculated as DEPS = (Zx + 0.5·Ax)/(Vx + Ax + Zx), where Zx = zeaxanthin, Ax = antheraxanthin, and Vx = violaxanthin (Kalituho et al. 2007a).

### 2.6 Statistical Analyses

Statistical analyses were performed in GraphPad Prism 10.1.0 (GraphPad Software, Boston, MA, USA). Normality of data distribution was tested with the Shapiro–Wilk test and by Q–Q plots; distribution of variance was inspected graphically (homoscedasticity plots). One‐way and two‐way analysis of variance (ANOVA) were used as appropriate. Specifically, a two‐way ANOVA was performed to evaluate the effects of applications, duration of incubation, light condition during incubation and their interactions with NPQ. When ANOVA *p* < 0.05, Dunnett's post hoc tests compared each antioxidant to the control. For dose–response curves, data were baseline‐corrected to the corresponding control and fitted with a polynomial model.

## 3 Results

Previous studies have shown that applying various antioxidants to plants affects their growth similarly, and non‐photochemical quenching (NPQ) differently (Rodrigues de Queiroz et al. 2023). However, these studies often used different plant species, application methods, concentrations, and growth conditions. This heterogeneity in experimental conditions makes it difficult to determine if distinct mechanisms underlie the observed variations in NPQ. In part because NPQ is highly sensitive to environmental factors such as light conditions (Demmig‐Adams et al. 2022), and natural genetic variation between and within species (Glowacka 2025).

### 3.1 Antioxidant Application Affects Photoprotection and Photochemistry

To investigate the effects of different antioxidants on photosynthetic parameters under similar conditions, 12‐day‐old *Arabidopsis thaliana* seedlings were treated with foliar sprays of the following antioxidants ascorbic acid (Asc), chitosan, coenzyme M (CoM), reduced glutathione (GSH), melatonin (Mel), and N‐acetyl cysteine (NAC). We initially applied high concentrations previously reported in the literature to maximise the likelihood of observing physiological effects. To control for differences in acidity between the diverse antioxidants tested, the pH of all solutions was adjusted to 5.1.

As an initial assessment of photosynthetic performance, we analysed *F*_*v*_/*F*_*m*_, and kinetics of ΦPSII and NPQ during 10 min of strong illumination followed by 10 min of darkness. These measures respectively indicate photosystem II (PSII) integrity, PSII light‐use efficiency during actinic illumination and dark recovery, and non‐photochemical energy dissipation under the same conditions. A key indicator of PSII function, the maximum quantum yield (*F*_*v*_/*F*_*m*_; Baker 2008) was unchanged in response to application of all antioxidants tested (Figure 1A). ΦPSII, which estimates the operating efficiency by which light absorbed by PSII is used to reduce quinone (*e.g*., photochemistry) and correlates with electron transport and CO_2_ assimilation efficiency (Baker 2008), was moderately effected by antioxidant application (Figure 1B). In contrast, NPQ kinetics showed clear and contrasting effects in response to multiple antioxidant applications (Figure 1C). Primary measurements are provided in Supporting Information Table S1.

We quantified ΦPSII and NPQ rates and plotted these and their maximums in light and darkness to better compare the observed changes. Melatonin slowed the rate of ΦPSII induction in the light by 38% (Figure 1D), leading to a 20% reduction in maximum ΦPSII by the end of the light period (Figure 1E). Conversely, NAC application increased the rate of ΦPSII induction by 37%, leading to an 18% increase in maximum ΦPSII. Upon returning to darkness, the ΦPSII induction rate increased by 75% with CoM and 53% with Mel, but decreased by 25% with NAC application (Figure 1F). The shifts in dark induction speed did not translate to meaningful differences ΦPSII at the end of the dark period (Supporting Information Table S1). This suggests a full recovery of ΦPSII across all treatments upon dark acclimation, consistent with our *F*_*v*_/*F*_*m*_ measurements.

For NPQ, NPQ maximum and the rates of its induction in light (NPQ Ind_Rate_) and relaxation in darkness (NPQ Rel_Rate_) were compared. Multiple antioxidant applications affected NPQ Ind_Rate_; CoM, GSH, and Mel decreased it by 39%, 21%, or 39%, respectively (Figure 1G). Maximum NPQ was decreased by CoM application (46%), but increased by applications of Chi (11%), Mel (10%) and NAC (19%; Figure 1H). NPQ Rel_Rate_ was decreased by CoM (86%), and increased by Chi (58%), Mel (160%) and NAC (52%; Figure 1I). No significant differences in the residual NPQ were found for any of the applications, as NPQ consistently returned to base levels within the 10 min recorded (Figure 1C). Overall, these results highlight that different antioxidant applications lead to divergent photosynthetic responses, particularly regarding NPQ kinetics.

### 3.2 Antioxidant Application Effects on NPQ are Concentration Dependent

The divergent responses observed at our initial antioxidant concentrations (Figure 1) raise the question of whether antioxidant effects on NPQ are inherently distinct or simply concentration‐dependent. We therefore evaluated a wider range of concentrations to determine the dose‐dependency of NPQ effects, ensuring a comprehensive comparison with existing literature.

**Figure 2:**
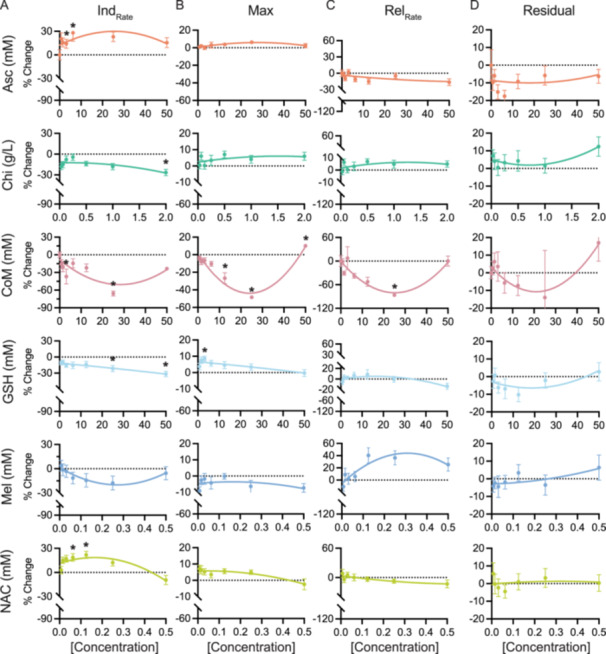
Dose‐dependency of applied antioxidants on non‐photochemical quenching (NPQ) kinetics. Twelve‐day‐old *Arabidopsis thaliana* plants were treated with pH‐buffered phosphate (control), or antioxidants at the concentrations indicated, ascorbic acid (Asc), chitosan (Chi), coenzyme M (CoM), reduced glutathione (GSH), melatonin (Mel), or N‐acetyl cysteine (NAC). NPQ was determined as in Figure 1. Filled circles are NPQ kinetics parameters derived as indicated in Figure 1C, lines shown are second‐order polynomial fit curves, error bars indicate standard error of the mean (*n* ≥ 19). Statistically significant differences between control and applications by Dunnett's Post Hoc Tests are indicated with asterisks (*p*‐value < 0.04). Percent change relative to control (dotted line) of A, NPQ induction rate (Ind_Rate_), B, maximum NPQ (Max), C, NPQ relaxation rate (Rel_Rate_) and D, residual NPQ. [Color figure can be viewed at wileyonlinelibrary.com]

Most antioxidant applications resulted in a dose‐dependent slowing of NPQ Ind_Rate_, with the exception of Asc and NAC, which showed increases at low concentrations (fastest rate for each was 30% and 22%, respectively; Figure 2A). The slowing of NPQ Ind_Rate_ was the most pronounced at 25 mM CoM, which caused a 66% decrease. Other antioxidant applications reduced NPQ Ind_Rate_ by only 32% (GSH) or 27% (Chi). For maximum NPQ (Figure 2B) and Rel_Rate_ (Figure 2C), most antioxidants had negligible effects. CoM was the exception: at 25 mM, it reduced maximum NPQ by 48% and reduced Rel_Rate_ by 86% (Figure 2B–C). Interestingly, the CoM effect on maximum NPQ reversed at higher concentrations, showing a 10% increase at 50 mM. NPQ_Res_ remained indistinuishable from control across all applications and concentrations tested. Primary measurements are provided in Supporting Information Table S2.

In general, these results confirm that antioxidant applications have distinct, concentration‐dependent effects on photoprotection. We concluded that the concentrations selected for our initial and subsequent comparative studies (centred on the X‐axes in Figure 2) are ideal for distinguishing maximal alterations of most NPQ parameters.

### 3.3 Antioxidant Application Effects on NPQ are Time and Light Dependent

Given that light history and the time of day can alter photosynthetic parameters (Ruban and Belgio 2014), we expanded our analysis to include multiple combinations of application incubation times and illumination. Specifically, we tested exposure time post‐antioxidant application between 30 min and 6 h, applying antioxidants at the same time of day. During the exposure time, seedlings were either illuminated or kept in darkness, after which they were dark adapted for 20 min and then NPQ parameters were determined. The effects of antioxidant application (A), time post‐antioxidant application (T) and their interaction (A x T) were evaluated, primary measurements are provided in Supporting Information Table S3.

**Figure 3:**
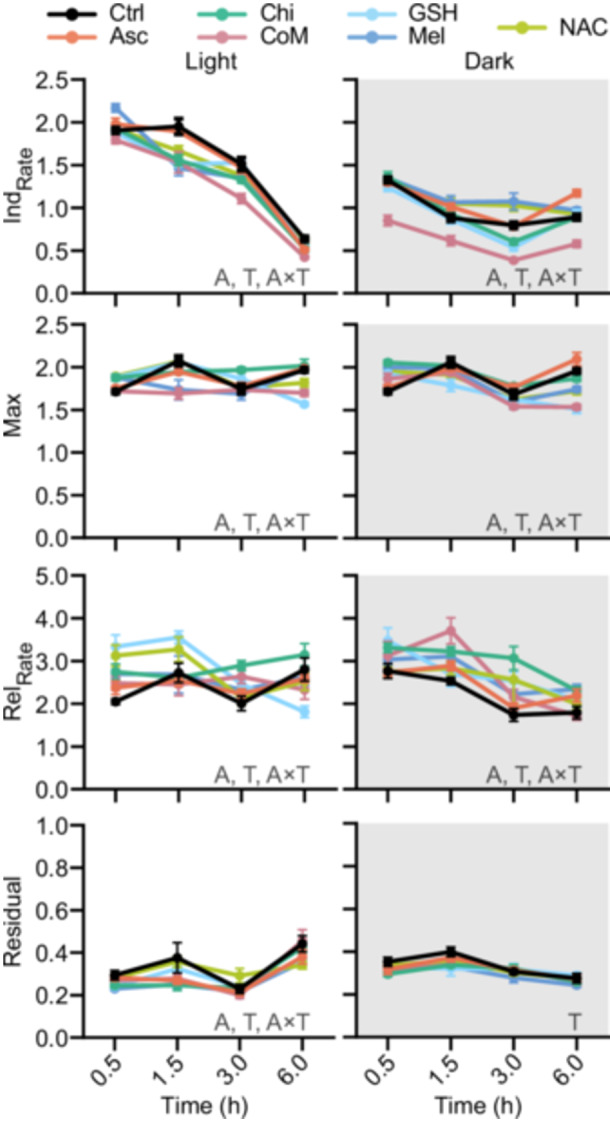
Post‐application duration and illumination modify antioxidant application effects on non‐photochemical quenching (NPQ) kinetics. 12‐day‐old *Arabidopsis thaliana* plants were treated with pH‐buffered phosphate (control), or pH‐buffered antioxidants at final concentrations of 25 mM ascorbic acid (Asc), 1 g/L chitosan (Chi), 25 mM coenzyme M (CoM), 25 mM reduced glutathione (GSH), 0.25 mM melatonin (Mel), or 0.25 mM N‐acetyl cysteine (NAC) and incubated for 0.5, 1.5, 3.0 or 6 h under illumination (Light) or darkness (Dark) prior to NPQ determination as in Figure 1. Filled circles are NPQ kinetics parameters derived as indicated in Figure 1C; lines connect the points for clarity, error bars indicate standard error of the mean, *n* ≥ 13. Induction rate of NPQ (Ind_Rate_), maximum NPQ (Max), relaxation rate of NPQ (Rel_Rate_) and residual NPQ are shown. Data were analysed with a two‐way ANOVA followed by Dunnett's Post Hoc Tests. Tested factors (A, antioxidant application; T, time; A×T, the interaction of A and T are indicated where they are significant (*p* < 0.02). Full statistical results are in Supporting Information Table S4 (two‐way ANOVA) and Supporting Information Table S5 (Dunnett's). [Color figure can be viewed at wileyonlinelibrary.com]

Exposure time post‐antioxidant application influenced all NPQ parameters significantly, with its strongest effect on Ind_Rate_ (*p*‐values < 0.0005, specific *p‐*values available in Supporting Information Table S4). Likewise, antioxidants applied affected NPQ post illumination or darkness incubation (*p*‐values < 0.004). The only NPQ parameter negligibly affected was NPQ_Res_ of plants incubated in darkness post‐application (Figure 3). Finally, there was a stark contrast between antioxidant exposure under illumination or darkess. For example, in all plants incubated with illumination after application, Ind_Rate_ decreased sharply over time, while for those incubated in darkness, Ind_Rate_ was more consistent over time.

Considering the effects of individual antioxidant applications, they were significant across all NPQ parameters except NPQ_Res_ after darkness, and were exposure time dependent (*p* < 0.0168; Figure 3, Supporting Information Table S4). Notably, several antioxidants had different effects on NPQ parameters at different times post‐application; pairwise statistics can be found in Supporting Information Table S5. For example, after application of GSH and dark incubation, maximum NPQ was increased at 30 min (*p* = 0.0168), but decreased at 1.5 h (*p* = 0.0004). Similarly, after application of CoM and dark incubation, Rel_Rate_ is indistinguishable from control at 30 min (*p* = 0.7) nor 3 h (*p* = 0.6), but it is increased at 1.5 h (*p* = 0.0009). Because the antioxidant applications had effects at all incubation timepoints, we selected the shortest time for the remainder of the experiments considering the effects were more likely to be direct at the shortest time. Regarding illumination conditions, darkness allowed more differentiation between antioxidant applications for NPQ rates. It is also likely to have simpler underlying physiology, as it separates active photosystems from antioxidant effects, thus we chose to continue using darkness.

In summary, our results suggest that the impact of antioxidants on NPQ is specific to the antioxidant used, independent of concentration (Figure 2), exposure time, and illumination conditions (Figure 3). This implies the existence of distinct molecular mechanisms underlying the action of each antioxidant.

### 3.4 Ascorbate and Coenzyme M Effects on NPQ are Dependent on Xanthophyll Cycle Enzymes

Based on our contrasting results for Asc and CoM treatments on NPQ kinetics (Figure 1) we performed further experiments to understand the underlying mechanisms responsible for the changes in NPQ kinetics. The majority of NPQ kinetics is explained at the molecular level by the xanthophyll cycle and structural changes in Photosystem II subunit S (PsbS) (Demmig‐Adams and Adams 2006). The xanthophyll cycle requires two enzymes, violaxanthin de‐epoxidase (VDE) and zeaxanthin epoxidase (ZEP). To investigate the role of these proteins in mediating the effects of Asc and CoM application on NPQ, we compared antioxidant applications on T‐DNA null mutants lacking PsbS (*psbs*), VDE (*vde*), ZEP (*zep*), or corresponding wild‐type plants.

**Figure 4:**
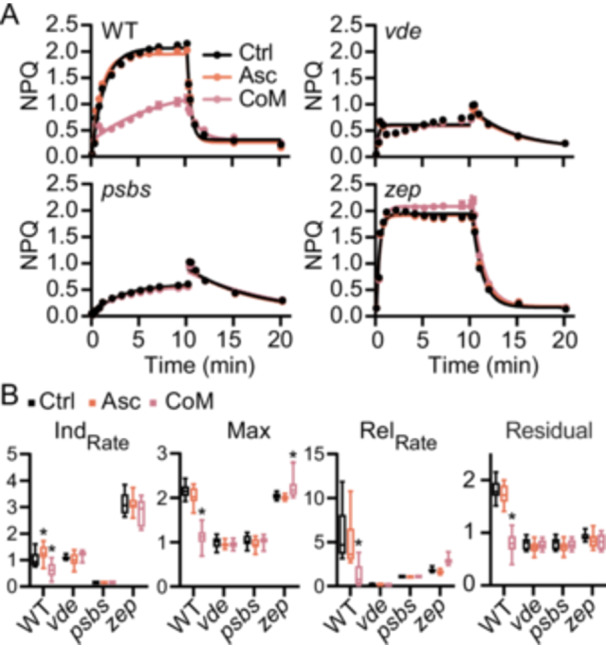
Contrasting effects of Asc and CoM application on non‐photochemical quenching (NPQ) kinetics in NPQ mutants. 12‐day‐old *Arabidopsis thaliana* plants (WT and *vde*, *psbs*, and *zep* null mutants) were treated with pH‐buffered phosphate (control), 25 mM ascorbic acid (Asc), or 25 mM coenzyme M (CoM). NPQ was determined as in Figure 1. A, NPQ kinetics over time during 10 min of illumination followed by 10 min of darkness for WT and NPQ mutants (*vde*, *psbs*, and *zep*). B, Induction rates of NPQ (Ind_Rate_) in illuminated conditions, calculated from the slope of the initial NPQ curve fit to a hyperbola, maximum NPQ in illuminated conditions (Max), relaxation rate of NPQ in the dark (Rel_Rate_), and residual NPQ during the final dark period. Boxes span 25th to 75th percentiles and whiskers indicate minimum and maximum values, *n* ≥ 10. Statistically significant differences between control and applications by Dunnett's Post Hoc Tests are indicated with asterisks (*p*‐value < 0.05). [Color figure can be viewed at wileyonlinelibrary.com]

The NPQ curves from the *vde*, *psbs*, and *zep* seedlings were distinct from wild type, irrespective of antioxidant (Figure 4A). Both *psbs* and *vde* mutants lowered NPQ maximums, while the *zep* mutant had more rapid NPQ induction (Figure 4A), consistent with previously reported phenotypes (Niyogi et al. 1998; Li et al. 2000; Kalituho et al. 2007a; Kalituho et al. 2007a). In contrast to wild‐type, the effects of Asc and CoM were attenuated or absent in the mutant backgrounds (Figure 4A), suggesting they do act through these major NPQ kinetic mechanisms. Primary measurements are provided in Supporting Information Table S6.

Parameterisation of the NPQ curves (Figure 4B) clarified the genotype‐dependent effects. As was previously reported, Asc increased NPQ Ind_Rate_ in wild type (Ivanov and Edwards 2000), in our hands by 22%. This was abrogated in every mutant background, suggesting that coordinated activity of all three proteins may be required for this enhancement. In contrast, CoM reduced Ind_Rate_ in wild type (39%), and also in the *zep* mutant (10%), but it had insignificant effects on Ind_Rate_ in *vde* or *psbs* mutants. Similarly, the CoM effects on other NPQ parameters remained distinct to the *zep* mutant. For maximum NPQ, CoM application caused a decrease in wild type (50%), and an increase (8%) in the *zep* mutant. The Rel_Rate_ was decreased by CoM application in wild type (76%) and increased insignificantly in the *zep* mutant. These observations suggest that the presence of PsbS and VDE is necessary for CoM‐mediated changes in NPQ kinetics.

Mechanistically, our observed changes in NPQ kinetics could be explained by 1) changes in abundance of PsbS, VDE, and ZEP proteins, 2) changes in lumen acidification affecting protein activity, or 3) direct modulation of xanthophyll cycle activity.

### 3.5 PsbS, VDE, and ZEP Abundances are Normal After Ascorbate and Coenzyme M Application

PsbS levels have been reported to correlate with maximum NPQ levels (Li et al. 2000; Peterson and Havir 2000; Turc et al. 2024). Similarly, the xanthophyll cycle depends on the abundance and activity of the enzymes VDE and ZEP (Niyogi et al. 1998). Therefore, we used immunodetection to quantify PsbS, VDE, and ZEP abundance post‐application of Asc or CoM under three conditions: dark (30 min of dark incubation), light (dark followed by 10 min of high light), and recovery (10 min of dark recovery after dark and light incubations) as indicated in Supporting Information Figure S1A. No significant differences were observed in any of the conditions tested (Supporting Figure S1B,C). Therefore, changes in NPQ kinetic parameters cannot be explained by differences in the abundance of these three proteins.

### 3.6 Lumen Acidification Appears Normal After Ascorbate and Coenzyme M Application

**Figure 5:**
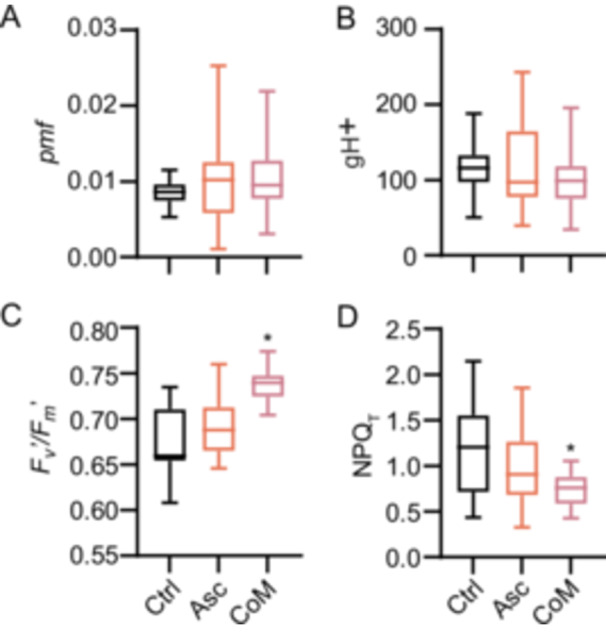
Effects of applied antioxidants on lumen acidification, quantum yield of PSII and theoretical NPQ. Three‐week‐old *Arabidopsis thaliana* plants were treated with pH‐buffered phosphate (control), 25 mM ascorbic acid (Asc), or 25 mM coenzyme M (CoM), dark‐adapted for 30 min then exposed to 5 min of high light (1000 µmol m^−2^s^−1^) prior to measurement. Boxes span 25th to 75th percentiles and whiskers indicate minimum and maximum values. Statistically significant differences between control and applications by Dunnett's Post Hoc Tests are indicated with asterisks. A, Trans‐thylakoid proton motive force (*pmf*, *n* ≥ 26). B, Proton flux (gH + , *n* ≥ 25). C, Maximum quantum yield of PSII (*F*_*v*_ */F*_*m*_) in light‐adapted plants (*n* ≥ 8, *p* = 0.001). D, Theoretical NPQ (NPQ_T_) in light‐adapted plants (*n* ≥ 26, *p* = 0.0002). [Color figure can be viewed at wileyonlinelibrary.com]

Changes in lumen acidification are the triggers for activating PsbS and VDE, and lack of acidification can lead to strong inhibition of NPQ (Demmig‐Adams and Adams 2006; Järvi et al. 2013). To gain insights into the level of lumen acidification on treated plants, we evaluated proton fluxes (gH^+^) and the trans‐thylakoid proton motive force (*pmf*) *in vivo*, by measuring electrochromic shift (ECS). For this experiment, antioxidants were applied to 3‐week‐old plants grown in soil, dark‐adapted for 30 min, and exposed to high light for 5 min prior to measurement. No significant differences in either *pmf* (Figure 5A) nor gH^+^ (Figure 5B) were observed in control versus treated plants, suggesting that they have comparable levels of lumen acidification. Interestingly, plants treated with CoM displayed an 9.6% increase in light‐adapted maximum quantum yield of Photosystem II (*Fv’/Fm’*), while Asc application had no significant effect (Figure 5C). Consistent with seedling data (Figures 1, 2, 3), in these experiments on 3‐week‐old plants, a simplified NPQ parameter reflecting the theoretical NPQ (NPQ_T_) (Tietz et al. 2017), was unaffected by Asc application and reduced by CoM application (38%; Figure 5D). In summary, lumen acidification and protein abundance both appeared to be normal upon light conditions inducing NPQ after Asc or CoM application.

### 3.7 Xanthophyll Cycle Dynamics Change in Response to Ascorbate and Coenzyme M Application

When illuminated, VDE converts violaxanthin to an antheraxanthin intermediate and then to zeaxanthin which allows dissipation of excess energy measured as NPQ (Demmig‐Adams and Adams 2006). Under low light, zeaxanthin is converted back to antheraxanthin and then violaxanthin by ZEP. This xanthophyll “cycle” balances light energy use and photoprotection (Jahns and Bethmann 2025).

**Figure 6:**
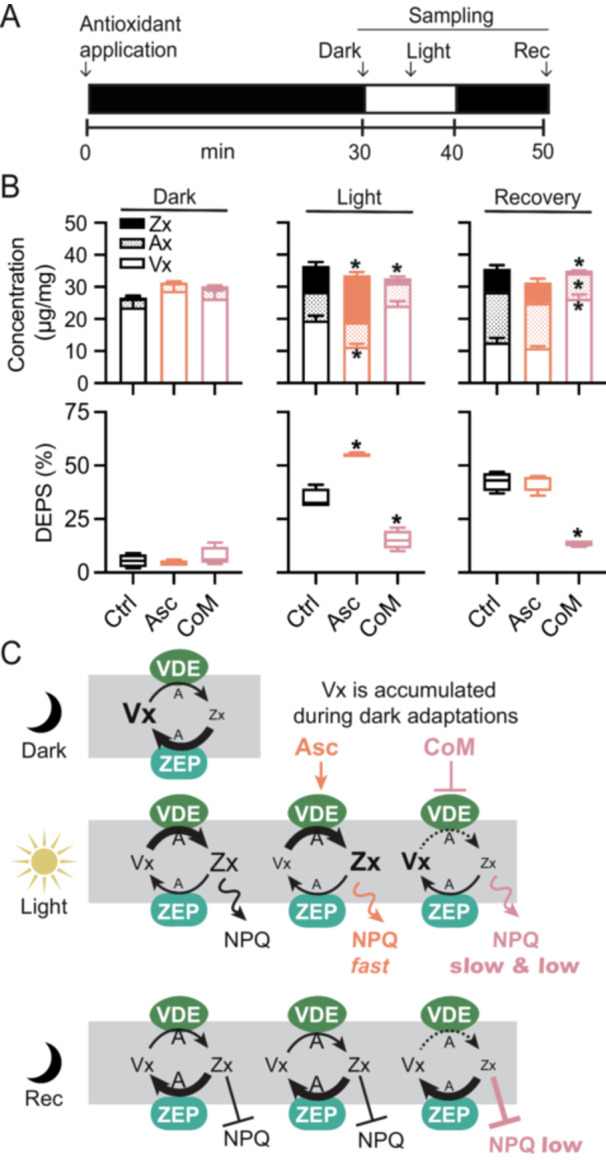
Effects of applied antioxidants on xanthophyll cycle component content in response to light perturbation. A, Schematic of experimental set‐up. Antioxidant application was performed at time 0, after 30 min of dark incubation, plants were exposed to 10 min of high light (1000 µmol m^−2^ s^−1^) followed by 10 min of dark recovery. Sampling occurred after dark incubation (Dark), during light (Light), and after 10 min of dark recovery (Recovery). B, Quantification of xanthophyll cycle pigments, violaxanthin (Vx, white bars), antheraxanthin (Ax, dotted bars), and zeaxanthin (Zx, solid bars) in plants treated with control (Ctrl; pH‐buffered phosphate), 25 mM ascorbic acid (Asc), or 25 mM coenzyme M (CoM) in samples collected at time points indicated in Figure 6A. Bars indicate means and standard error of the mean (*n* = 4 biological replicates). Corresponding raw data are shown in Supporting Information Table S7. De‐epoxidation states (DEPS) of the xanthophylls in the same samples are shown as a percentage (%). Boxes span 25th to 75th percentiles and whiskers indicate minimum and maximum values. Statistically significant differences between control and applications by Dunnett's Post Hoc Tests are indicated with asterisks (*p* < 0.009). C, Schematic model of application of Asc or CoM interacting with the light‐dependent interconversion of Vx, Ax, and Zx. Violaxanthin de‐epoxidase (VDE) removes epoxy groups from Vx to form Ax then Zx. To complete the cycle, Zx is re‐epoxidized by zeaxanthin epoxidase (ZEP) to Vx, again through an Ax intermediate. Relative levels of Vx, and Zx are indicated by the size and thickness of their letters, and relative flux by the size and thickness of the arrows. Dark and light conditions are indicated by “crescent moon” or “sun” symbols, respectively. In darkness, accumulation of Vx is favoured, while in high light accumulation of Zx is favoured. Zx stimulates non‐photochemical quenching (NPQ). Application of Asc increases VDE activity, seen in the high levels of Zx in light, stimulating NPQ. CoM application decreases VDE activity, seen in reduced turnover of Vx to Zx in light, lowering NPQ. [Color figure can be viewed at wileyonlinelibrary.com]

To determine if Asc or CoM applications directly influence the xanthophyll cycle, we measured its pigments post‐application of Asc or CoM under three conditions intended to mimic the light treatments used to measure NPQ: dark (30 min of dark incubation), light (dark followed by 10 min of high light), and recovery (10 min of dark recovery after dark and light incubations) as indicated in Figure 6A.

Under dark‐adapted conditions, xanthophyll cycle pigment levels and their total de‐epoxidation state (DEPS, ~5%) remained consistent across all treatments (Figure 6B, Dark). In the light, VDE activation in control plants increased zeaxanthin and antheraxanthin levels at the expense of violaxanthin, reaching a DEPS of approximately 35% (Figure 6B, Light). Asc application enhanced this transition, increasing zeaxanthin by 81% and DEPS by 60% relative to the control, consistent with its role as a VDE substrate (Foyer and Kunert 2024).

In contrast, CoM application suppressed light‐driven de‐epoxidation, resulting in 81% less zeaxanthin accumulation and a 56% lower DEPS compared to the control. During the transition to dark recovery, ZEP was activated in control and Asc‐treated plants, leading to similar antheraxanthin increases and DEPS of 43%. However, CoM‐treated plants exhibited altered flux, maintaining 82% less zeaxanthin and a DEPS 68% lower than the control (Figure 6B, Recovery). Notably, total xanthophyll cycle pigment and chlorophyll concentrations, lutein levels, and chlorophyll a/b ratios remained unchanged across all conditions (Supporting Information Table S7), proving that observed changes to NPQ are not from de novo pigment synthesis. Collectively, these results are consistent with the conclusion that the major influence of Asc and CoM are on xanthophyll cycle activity by exerting opposing effects on VDE activity.

## 4 Discussion

Exogenous antioxidants are frequently used to mitigate oxidative stress in plants, yet their reported effects on photosynthetic performance have been inconsistent across species and experimental conditions. By applying six antioxidants at a standardised pH, and with multiple dosing and illumination regimes, we demonstrate that these compounds do not exert a uniform effect on photoprotection. Instead, they drive compound‐specific and context‐dependent divergent shifts in NPQ induction, maximum capacity, and relaxation (Figures 1C,G–I and 2, 3, 4), which do not strictly parallel observed changes in ΦPSII (Figure 1A,B,D–F). These results underscore the high sensitivity of photoprotective energy dissipation to exogenous redox‐active compounds. Because NPQ kinetics and amplitude directly influence light‐use efficiency and productivity, particularly in fluctuating light environments (Hubbart et al. 2012; Kromdijk et al. 2016; Sahay et al. 2023), our data suggest that exogenously applied antioxidants could be leveraged to tune photon use in both experimental and field settings.

### 4.1 NPQ Responses to Antioxidants Depend Strongly on Dose, Time, and Light History

We found that NPQ parameters varied substantially with antioxidant concentration (Figure 2), incubation time, and light conditions after application (Figure 3). This context dependence likely contributes to the divergent outcomes reported previously, where plant species, delivery method, concentration, and illumination regimes differ (Hasanuzzaman et al. 2019; Rodrigues de Queiroz et al. 2023). The robustness of our findings is supported by consistent internal replication across multiple experimental iterations (Figures 1, 2, 3). Notably, the most potent treatments, frequently Coenzyme M, exhibited highly reproducible and statistically significant effects on NPQ kinetics across all trials. In contrast, some treatments always resulted in high variability. For example, the effect of melatonin on NPQ Rel_Rate_ is the same in Figures 1 and 2, but the variability of biological replication resulted in different statistical significance (Figure 1 *p* < 0.0001, vs*.* Figure 2 *p* = 0.09 at the same concentration). These instances likely underscore the sensitivity of that antioxidant's mechanistic interaction with NPQ. Melatonin application was previously shown to increase maximum NPQ in tomato seedlings under chilling stress (Ding et al. 2017) or decrease maximum NPQ following application in maize (Chen et al. 2018; Huang et al. 2019). And NPQ itself is an exquisitely sensitive process shaped by light history and diel physiology (Kalituho et al. 2007a). Consistently, we observed that NPQ levels varied with incubation time and were strongly influenced by pre‐illumination conditions (Figure 3). These results reinforce the need for careful experimental design when comparing antioxidant effects on NPQ and suggest that antioxidant–NPQ relationships are best interpreted in their environmental context.

### 4.2 Ascorbate Accelerates NPQ Through the Xanthophyll Cycle

Asc was distinctive in accelerating NPQ induction (Figures 1, 2, 4), which aligns well with its known role as a substrate for VDE (Bratt et al. 1995). This is further supported by the opposite effect observed in mutants with reduced Asc levels (Müller‐Moulé et al. 2002; Kappel et al. 2025). A mechanistic explanation is straightforward: Asc is a substrate for VDE, donating electrons required for violaxanthin de‐epoxidation to zeaxanthin (Bratt et al. 1995). Accordingly, increasing Asc availability is expected to enhance VDE activity and thereby increase zeaxanthin accumulation under high light (Figure 6C). Our pigment profiling supports this model, as Asc‐treated plants showed elevated zeaxanthin and an increased de‐epoxidation state (DEPS) in high light relative to controls (Figure 6B). This appears to be the primary effect of Asc 30 min after application, as its effects were abrogated by xanthophyll cycle mutants (Figure 4). Moreover, Asc did not affect lumen acidification as measured by electrochromic shift (Takizawa et al. 2007; Figure 5) or xanthophyll cycle protein levels (Supporting Information Figure S1). Together, these results provide a consistent biochemical link between exogenous Asc and faster engagement of xanthophyll‐cycle–dependent NPQ.

### 4.3 Coenzyme M Inhibits NPQ Through the Xanthophyll Cycle

In contrast to Asc, the non‐native archaeal antioxidant CoM caused the strongest inhibition of NPQ, decreasing maximum NPQ, and slowing induction and relaxation rates (Figures 1, 2, 3, 4). Notably, chitosan is also non‐native to plants, yet it did not trigger CoM‐like NPQ inhibition, suggesting that non‐native antioxidants interact distinctly with photoprotection. Our model of CoM application mechanism is that it suppresses VDE activity shortly after application (Figure 6C). CoM almost entirely blocked light‐driven zeaxanthin accumulation, despite stable baseline pools of xanthophyll pigments (Figure 6B). This inhibitory effect appears to be the primary driver of CoM action, as its effects were dependent on the presence of VDE and PsbS, but not ZEP (Figure 4). Parallel to the Asc results, CoM's major effect was on the xanthophyll cycle activity, with no detectable affect on lumen acidification measured by electrochromic shift (Figure 5) or the abundance of PsbS, VDE, and ZEP proteins (Supporting Information Figure S1). Together, these results are consistent with xanthophyll‐cycle suppression as the biochemical basis for CoM‐mediated NPQ inhibition.

### 4.4 Why do Thiol‐Containing Antioxidants Diverge in Their NPQ Effects?

It is tempting to suggest that thiol‐containing antioxidants behave similarly. The thiol reductant dithiothreitol is a well‐known inhibitor of NPQ (Bilger et al. 1989; Bailey and Walker 1992; Neubauer 1993) that can severely decrease maximum NPQ through direct inhibition of VDE (Yamamoto and Kamite 1972; Hallin et al. 2015). Indeed, CoM also contains a thiol and its effects on NPQ mimic direct inhibition of VDE, as they strongly suppress light‐driven violaxanthin de‐epoxidation (Figure 6B). However, our broader antioxidant panel indicates that the presence of thiol chemistry alone does not predict NPQ outcomes. Both GSH and NAC contain thiols, yet they exhibited different effects on NPQ kinetics. GSH‐treated plants were generally similar to controls for maximum NPQ and kinetic rates (Figure 1C,G–I), while NAC increased NPQ relaxation rates and its maximum (Figure 1H–I). The NAC increases to maximum NPQ are notable as they are also opposite of the effects of CoM and dithiothreitol (Neubauer 1993). Even when multiple concentrations are considered, only GSH and NAC look similar in their effects on NPQ (Figure 2), though they continue to differ in induction rate. The contrasting NPQ signatures of thiol‐containing reductants emphasise that even at our shortest time point, uptake, compartmentation, reactivity, and specific molecular targets likely differ, emphasising the value of mechanistic and comparative investigations.

### 4.5 Limitations and Future Directions

This study establishes a standardised application process and provides mechanistic dissection for two contrasting antioxidants (Asc and CoM), but several questions remain. First, longer‐term experiments and fluctuating‐light assays will be important to connect kinetic changes to growth and stress outcomes, consistent with the productivity relevance of NPQ dynamics (Kromdijk et al. 2016). For example, while here we observed decreases in NPQ induction, relaxation, and maximum capacity 30 min after CoM application (Figures 1, 2, 3, 4), in tobacco, we previously showed that 24 h post‐application, NPQ induction, relaxation, and maximum capacity were actually increased (Brown et al. 2025). This reversal implies that the timescale of incubation is a critical variable in predicting outcomes. Second, CoM's precise molecular targets remain unresolved. Direct biochemical assays of VDE activity with redox manipulation could test whether CoM inhibits VDE directly, and redox profiling of antioxidant‐treated chloroplasts could determine whether it interacts indirectly through a redox signalling pathway. Finally, extending mechanistic profiling across the remaining antioxidants will help reveal whether they tune NPQ primarily through xanthophyll‐cycle dynamics, antenna reorganisation, signalling‐mediated acclimation, or other redox‐sensitive processes.

## 5 Conclusions

In summary, exogenous antioxidants exert specific, context‐dependent effects on NPQ. 30 min after application, Asc accelerates NPQ through its established role as a VDE substrate, increasing light‐driven violaxanthin de‐epoxidation and zeaxanthin accumulation. CoM, in contrast, inhibits the induction and relaxation of NPQ and achieves lower maximum protection. It requires VDE and PsbS for its effect and suppresses light‐induced violaxanthin de‐epoxidation while leaving protein abundance and *pmf* formation unchanged, consistent with inhibition of xanthophyll‐cycle activity.

## Supporting information


**Figure S1:** Protein levels of non‐photochemical quenching components are not altered by Asc or CoM applications.

**Table S1:** Chlorophyll fluorescence data presented in Figure 1. QY refers to operation efficiency of photosystem II, NPQ refers to non‐photochemical quenching, L1 to L12 and Lss refers to light points, and D1 to D6 refers to dark points.
**Table S2:** Chlorophyll fluorescence data presented in Figure 2. QY refers to operation efficiency of photosystem II, NPQ refers to non‐photochemical quenching, L1 to L12 and Lss refers to light points, and D1 to D6 refers to dark points.
**Table S3:** Chlorophyll fluorescence data presented in Figure 3. QY refers to operation efficiency of photosystem II, NPQ refers to non‐photochemical quenching, L1 to L12 and Lss refers to light points, and D1 to D6 refers to dark points.
**Table S4:** Two‐way ANOVAs for data presented in Figure 3.
**Table S5:** Dunnett's Post Hoc multiple comparison tests for data presented in Figure 3.
**Table S6:** Chlorophyll fluorescence data for non‐photochemical quenching presented in Figure 4.
**Table S7:** Quantification of photosynthetic pigments in leaf tissue in μg/mg presented in Figure S6, where a indicates dark, b high light, and c recovery.

## Data Availability

The data that supports the findings of this study are available in the supportingmaterial of this article.
