# Supplementary material for: Antioxidants Differentially Regulate Non‐Photochemical Quenching
Source: Plant Cell Environ. 2026 Jun 15;49(9):6960–73. doi: 10.1111/pce.70672 (PMC13436460; doi:10.1111/pce.70672)
Supplement: Supplementary file 1 [file PCE-49-6960-s002.pdf]

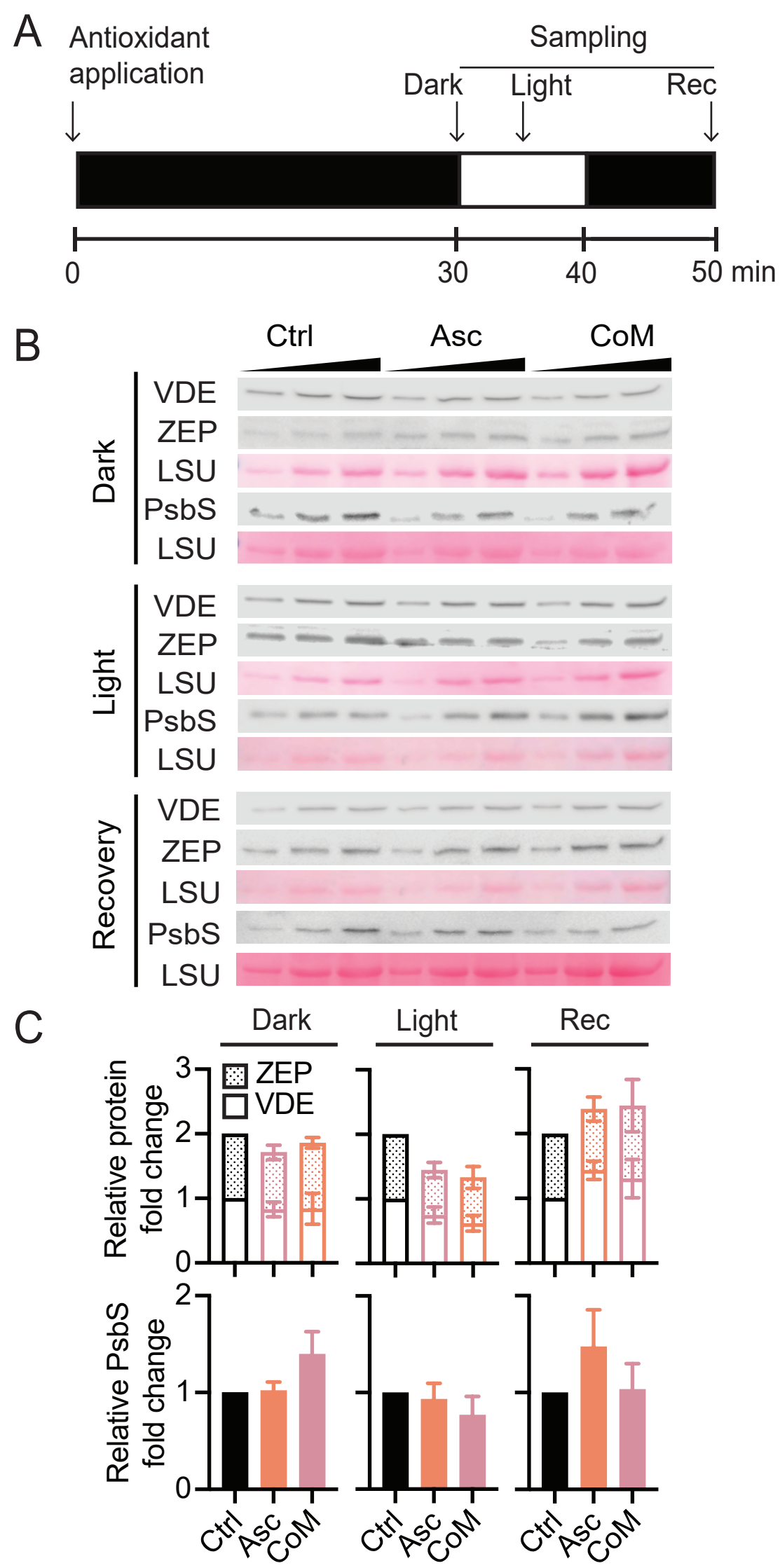

Supplemental Figure 1. Protein levels of nonphotochemical quenching components are not altered by Asc or CoM treatments.
A) Schematic of experimental set-up. Antioxidant application was performed at time 0, after 30 minutes of dark incubation, plants were exposed to 10 minutes of high light (1000 µmol m-2s-1) followed by 10 minutes of dark recovery. Sampling occurred after dark incubation (Dark), during high light (Light), and after 10 minutes of dark recovery (Res). B) Immunoblots of Violaxanthin de-epoxidase (VDE), Zeaxanthin epoxidase (ZEP), and corresponding total protein by visualizing RUBISCO large subunit (LSU) by Ponceau S stain. Immunoblots of Photosystem II Subunit S (PsbS) and corresponding total protein with Ponceau stain (LSU); n = 3. C) Quantification of protein abundance as a ratio of control levels in each condition. Protein electrophoresis for VDE and ZEP was performed with 10% gels and 15% for PsbS, amounts of total protein were 10, 20, and 30 ug for each treatment. There are no significant differences between control and Asc and CoM treatments.
